# Prenatally diagnosed submicroscopic familial aberrations at 18p11.32 without phenotypic effect

**DOI:** 10.1186/1755-8166-4-27

**Published:** 2011-12-02

**Authors:** Malgorzata I Srebniak, Marjan Boter, Carla MA Verboven-Peerden, Gerda AG Looye-Bruinsma, Gretel Oudesluijs, Robert-Jan H Galjaard, Diane Van Opstal

**Affiliations:** 1 Department of Clinical Genetics, Erasmus Medical Center, Rotterdam, Dr Molewaterplein 50, 3015 GE, the Netherlands

**Keywords:** 18p11.32 deletion, 18p11.32 duplication, submicroscopic abnormality, normal phenotype, MLPA, SNP array

## Abstract

**Background:**

Recent development of MLPA (Multiplex-Ligation-dependent Probe Amplification, MRC-Holland) and microarray technology allows detection of a wide range of new submicroscopic abnormalities. Publishing new cases and case reviews associated with both clinical abnormalities and a normal phenotype is of great value.

**Findings/results:**

We report on two phenotypically normal foetuses carrying a maternally-inherited interstitial submicroscopic abnormality of chromosome 18p11.32. Both abnormalities were found with the aneuploidy MLPA kit P095 during rapid aneuploidy detection, which was offered along with conventional karyotyping. Foetus 1 and its mother have a 1,7 Mb deletion and foetus 2 and its mother have a 1,9 Mb duplication. In both cases normal babies were born. We used the HumanCytoSNP-12 array of Illumina to visualize the CNVs and map the breakpoints.

**Conclusions:**

We suggest that a CNV at 18p11.32 (528,050-2,337,486) may represent a new benign euchromatic variant.

## Findings

Partial 18p monosomy and 18p trisomy both refer to a chromosomal disorder resulting from the absence or duplication of a part of the short arm of chromosome 18. Clinical features of monosomy 18p include mild to moderate mental retardation, short stature, round face with short protruding philtrum, palpebral ptosis and large ears with detached pinnae [1]. Familial transmission of partial monosomy 18p is rare and has only been reported in a few cases [2]. 18p trisomy is a rare finding and is often associated with a quite mild and nonspecific phenotype, even when the whole arm is duplicated. Most of the patients have either an apparently normal phenotype or minor anomalies, and may or may not have mental retardation [3]. Most of the published cases have cytogenetically visible abnormalities of 18p. Recent development of MLPA (Multiplex-Ligation-dependent Probe Amplification, MRC-Holland) and microarray technology allows detection of a wide range of submicroscopic abnormalities. The application of these techniques can reveal new microdeletions/microduplications of unknown clinical relevance. Publishing new cases and case reviews associated with both clinical abnormalities and a normal phenotype is of great value.

In the present study we report on two prenatally detected cases of familial aberrations at 18p11.32 associated with a normal phenotype.

### Case 1

A 38 year-old Gravida 2, Para 1 was referred for prenatal cytogenetic diagnosis because of advanced maternal age. Amniocentesis was performed at 17 weeks of gestation and rapid aneuploidy detection (RAD) by using MLPA kit P095 was offered along with conventional karyotyping. RAD on uncultured amniotic fluid cells showed a diminished signal from a terminal probe on 18p (the *TYMS *gene). The parents were informed about the MLPA finding and parental blood was sampled immediately to perform parental studies (FISH) simultaneously with prenatal karyotyping. The deletion was confirmed by using fluorescent in situ hybridization (FISH) with probe RP11-145B19 overlapping the *TYMS *([MIM:188350]) gene. The karyotype in cultured amniotic fluid cells was 46,XX. FISH on parental chromosomes revealed that the mother carried the same deletion. The prenatal diagnosis and the parental studies (FISH) were completed within 17 days.

As the phenotype of both the mother and the foetus (the second trimester ultrasound) was normal, the parents decided to continue the pregnancy.

A healthy baby girl was born at term. She is now 2,5 years old (no dysmorphic features) and there are no indications for developmental delay or mental retardation.

**Figure 1:**
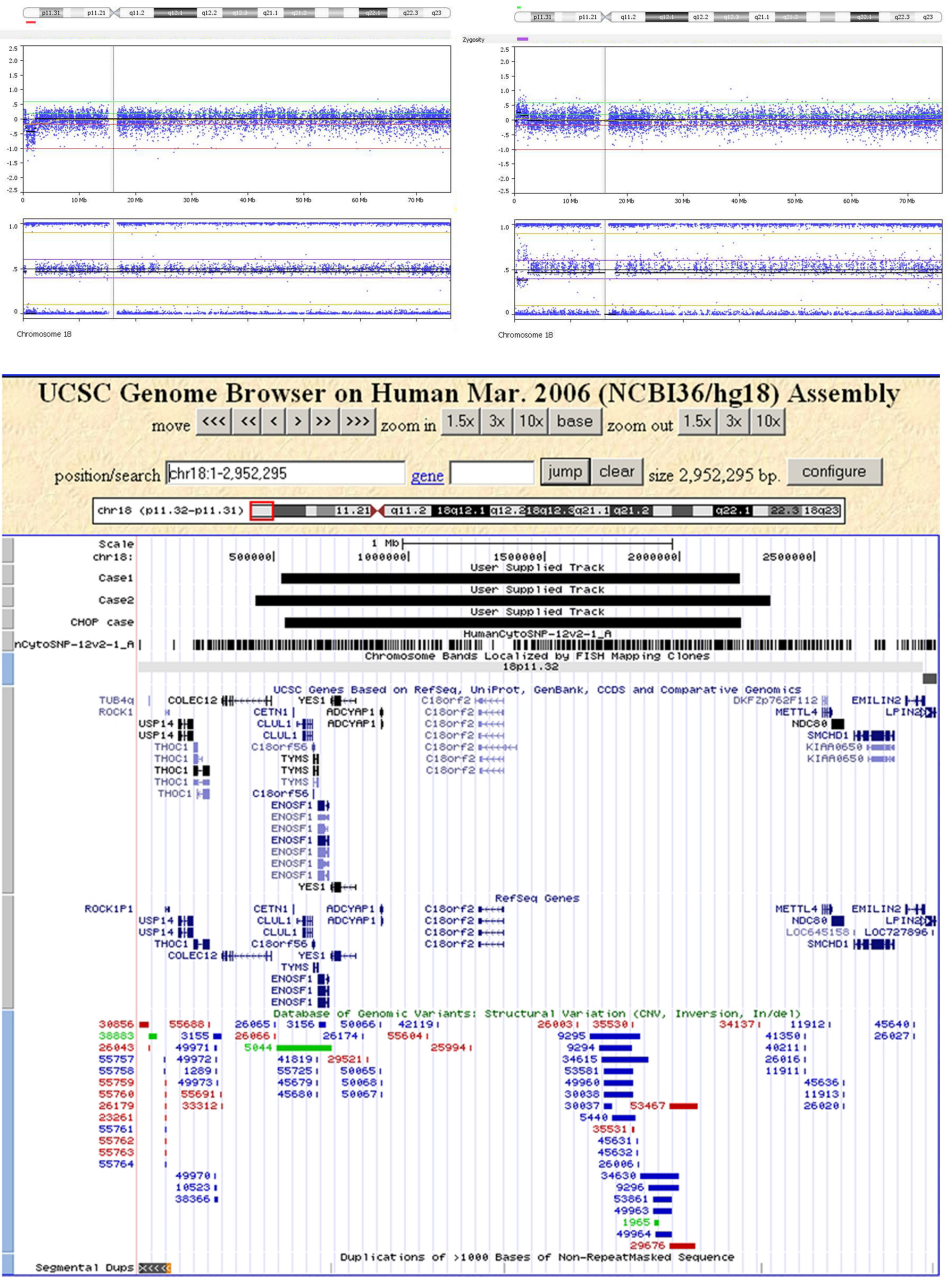
**18p11.32 abnormalities in cases 1, 2 and CHOP patient**. Left upper panel shows array results of case 1: chromosome 18 plot with 1,7 Mb interstitial deletion of 18p11.32. Right upper panel presents array results of case 2: chromosome 18 plot with 1,9 Mb interstitial duplication of 18p11.32. Lower panel shows the results in UCSC genome browser view (Hg18), gene content of the affected region and a comparison of case 1, case 2 en CHOP case. Variations listed on DGV are visible below the reference sequences.

To visualize the deletion and to map the breakpoints a targeted array analysis (HumanCytoSNP-12 of Illumina) was performed in a research setting according to the manufacturer instructions and analyzed by using Nexus Copy Number 5.0 (BioDiscovery software) (UCSC Mar. 2006 (NCBI36/hg18)). The array confirmed a deletion of 1,7 Mb and the foetal karyotype was revised: 46, XX.arr 18p11.32(528,050-2,226,095)x1 mat. Figure 1 left upper panel shows the array result of chromosome 18.

### Case 2

A 28 year-old woman (Gravida 1, Para 1) was referred to our academic hospital for first trimester prenatal screening and advanced ultrasound screening after term provision of the pregnancy which showed an increased NT (Nuchal Translucency) and echo-lucent structure in thorax. At 11+4 weeks of gestation a herniation of abdominal contents into the proximal part of the umbilical cord and a hydrothorax was seen. At 14+2 weeks of gestation small jugular sacs were seen. The hydrothorax and herniation into the umbilical cord were resolved. Amniocentesis was performed at 16 weeks of gestation and RAD by using MLPA kit P095 was offered along with conventional karyotyping and screening for 22q11 deletion (DiGeorge Syndrome (DGS), [MIM:188400]) (MLPA kit P250). MLPA for 22q11 was normal. RAD on uncultured amniotic fluid showed an enhanced signal from a terminal probe on 18p (the *TYMS *gene). The parents were informed about the MLPA finding; parental blood was sampled immediately to perform parental studies simultaneously with prenatal karyotyping. The karyotype in cultured amniotic fluid cells was normal. An inter- and intrachromosomal insertion was excluded using FISH with probe RP11-145B19. MLPA on parental DNA revealed that the mother was a carrier of the same duplication. The prenatal diagnosis and the parental studies were completed within 24 days. The 1,9 Mb gain was confirmed by performing targeted array (HumanCytoSNP-12 of Illumina), analyzed by using Nexus Copy Number 5.0 (BioDiscovery software) (UCSC Mar. 2006 (NCBI36/hg18). The revised foetal karyotype was 46, XY.arr 18p11.32(431,574-2,337,486)x3 mat. The parents were also informed about the targeted array-testing, which was then reported 20 days later. In order to exclude Noonan syndrome ([MIM:163950]) (increased NT) additional tests were performed. The DNA-analysis (*PTPN11 *([MIM:176876]), *RAF1 *([MIM:164760]), *SOS1 *([MIM:182530]) and *KRAS *([MIM:190070])) showed no mutations. Second trimester ultrasound screening at 20+3 gestational weeks showed no foetal abnormalities. As the phenotype of both the mother and the foetus (the second trimester ultrasound) was normal, the parents decided to continue the pregnancy. Ultrasound screening at 32+3 also was normal. A healthy baby boy was born at term. Figure 1 right upper panel shows the array results of chromosome 18.

In 2004-2010 we have performed RAD by using MLPA kit P095 on 5764 patients (4000 [4] + 1764 samples August 2007- November 2010). These were the only two cases which showed an abnormal result with the probe specific for the *TYMS *gene only.

If abnormal results for the most proximal or distal chromosome regions are obtained during RAD by using MLPA or QF-PCR, they may represent clinically significant regional imbalance. These findings should be detailed in the report and further testing is recommended [5].

We report on two such cases: phenotypically normal foetuses carrying a maternally-inherited interstitial submicroscopic abnormality of chromosome 18p. Foetus 1 and its mother have a 1,7 Mb deletion and foetus 2 and its mother have a 1,9 Mb duplication. According to the UCSC human genome database the abnormal region presented here encompasses 8 genes: *CETN1 *([MIM:603187]), *CLUL1*, *C18orf56*, *TYMS*, *ENOSF1 *([MIM:607427]), *YES1 *([MIM:164880]), *ADCYAP1 *([MIM:102980]) and *C18orf2 *([MIM:606486]). Additionally the duplication (case 2) involves also *COLEC12 *([MIM:607621]). None of these genes is associated with a known genetic syndrome.

Only a few cases with a pure de novo 18p monosomy are described [6,7]. Most of the trisomy 18p cases are not isolated, being associated with proximal trisomy 18q and other segmental 18q imbalances. Other cases are associated with an imbalance of another chromosome resulting from the segregation of a parental translocation [3]. Correlation between genotype and phenotype when a second chromosome is involved seems to have a limited value as the second chromosome may also contribute to the phenotype [8]. There are even fewer familial cases in the literature: 8 cases of familial deletion 18p [2]. However all these patients had microscopically visible abnormality. In cases not analysed by array, it is possible that the differences in phenotype can be explained by the gene content due to different breakpoints. If the cases were not tested with a molecular technique, it might be even difficult to distinguish between interstitial and terminal aberrations.

Submicroscopic 18p deletions were already reported. Recently published cases with abnormal phenotype that were tested with array involve larger terminal abnormalities [9,10]. Terminal deletions found by using subtelomeric FISH probe or subtelomeric MLPA are also difficult to compare with our patient who has an interstitial submicroscopic aberration and normal subtelomeric regions (Figure 1) [11].

In case of such subtle abnormalities we search our own databases, Toronto Database of Genomic Variants (DGV) (http://projects.tcag.ca/variation/), Children's Hospital of Philadelphia (CHOP) database (http://cnv.chop.edu) [12], Decipher (http://decipher.sanger.ac.uk), ISCA database (https://www.iscaconsortium.org/) and literature looking for similar cases in a normal and abnormal population. We have found one normal individual with a deletion of comparable breakpoints and size and a normal phenotype in CHOP database. We believe that the child from CHOP database with a 1,68 Mb deletion (chr18:541,688-2,230,220) together with our unaffected cases (2 children and their mothers) represents the fifth healthy individual with an interstitial abnormality of the same region on 18p. Figure 1 lower panel shows the three CNVs: case 1 (deletion 18p11.32), case 2 (duplication 18p11.32), the CHOP European individual (deletion 18p11.32) and the gene content of the affected region.

There are also a few comparable cases in Decipher database, which overlap with our patients. Patients 257164 and 253425 respectively have a 0,07 Mb gain and 0,51 Mb gain inherited from a normal parent, which supports our theory that a CNV within 18p11.32 (528,050-2,337,486) is most probably a benign variation. However there are also two other patients with an abnormal phenotype one with a 1,29 Mb gain of unknown inheritance (Decipher 253424) and one with a de novo 1,18 Mb loss (Decipher 257509). Whether 18p CNV is the only genomic aberration in these patients is unclear. Although 18p is not yet known as imprinted region the parental origin of the CNV might also explain the variability of the phenotype. In our cases both abnormalities were inherited from a normal mother.

Because there is only one case of a patient with an abnormal phenotype and a de novo CNV and in total 7 normal individuals (4 current cases, 1 CHOP and 2 Decipher normal parents) carrying a CNV within 18p11.32 (528,050-2,337,486) we suggest that a CNV in this region may represent a new benign euchromatic variant.

## Consent

Patients undergoing prenatal diagnosis at our medical university are informed that we may investigate (publish) their medical data as long as all data remained anonymised. Each patient had the opportunity to object to their inclusion within the published data. No objections were made to this publication.

## Competing interests

The authors declare that they have no competing interests.

## Authors' contributions

MIS coordinated the study, wrote the paper and studied the literature. MB performed the MLPA and microarray analyses. CAMVP and GAGLB performed cytogenetic analyses. GO did the genetic counselling of the parents, coordinated by R-JHG. MIS, R-JHG and DVO were responsible for the final (molecular) cytogenetic diagnoses and reports. All authors read and approved the manuscript.

## References

[B1] Turleau C Monosomy 18p Orphanet J Rare Dis 2008 3 4 10.1186/1750-1172-3-4 18284672 PMC2265258

[B2] Misceo D Orstavik KH Lybaek H Sandvig I Ormerod E Houge G Frengen E Inheritance of a terminal 7.1 Mb 18p deletion flanked by a 2.3 Mb duplication from a physically normal mother Am J Med Genet A 2009 149A 2877 2881 10.1002/ajmg.a.33106 19938092

[B3] Marical H Le Bris MJ Douet-Guilbert N Parent P Descourt JP Morel F De Braekeleer M 18p trisomy: a case of direct 18p duplication characterized by molecular cytogenetic analysis Am J Med Genet A 2007 143A 2192 2195 10.1002/ajmg.a.31881 17676610

[B4] Van Opstal D Boter M de Jong D van den Berg C Bruggenwirth HT Wildschut HI de Klein A Galjaard RJ Rapid aneuploidy detection with multiplex ligation-dependent probe amplification: a prospective study of 4000 amniotic fluid samples Eur J Hum Genet 2009 17 112 121 10.1038/ejhg.2008.161 18781187 PMC2985961

[B5] Professional Guidelines for clinical cytogenetics and clinical molecular genetics: QF-PCR for the Diagnosis of Aneuploidy Best Practise Guidelines http://www.cytogenetics.org.uk/

[B6] Taine L Goizet C Wen ZQ Chateil JF Battin J Saura R Lacombe D 18p monosomy with midline defects and a de novo satellite identified by FISH Ann Genet 1997 40 158 163 9401105

[B7] Tonk V Krishna J Case report: denovo inherited 18p deletion in a mother-fetus pair with extremely variable expression, confirmed by fluorescence in situ hybridization (FISH) analysis Eur J Obstet Gynecol Reprod Biol 1997 73 193 196 10.1016/S0301-2115(97)02749-8 9228504

[B8] Brenk CH Prott EC Trost D Hoischen A Walldorf C Radlwimmer B Wieczorek D Propping P Gillessen-Kaesbach G Weber RG Engels H Towards mapping phenotypical traits in 18p- syndrome by array-based comparative genomic hybridisation and fluorescent in situ hybridisation Eur J Hum Genet 2007 15 35 44 10.1038/sj.ejhg.5201718 17024214

[B9] Abu-Amero KK Hellani A Salih MA Alorainy IA Zidan G Kern KC Sicotte NL Bosley TM Optic disk and white matter abnormalities in a patient with a de novo 18p partial monosomy Ophthalmic Genet 2010 31 147 154 10.3109/13816810.2010.492817 20565246

[B10] Zavala J Ramirez M Medina R Heard P Carter E Crandall A Hale D Cody J Escamilla M Psychiatric syndromes in individuals with chromosome 18 abnormalities Am J Med Genet B Neuropsychiatr Genet 2010 153B 837 845 19927307 10.1002/ajmg.b.31047

[B11] Babovic-Vuksanovic D Jenkins SC Ensenauer R Newman DC Jalal SM Subtelomeric deletion of 18p in an adult with paranoid schizophrenia and mental retardation Am J Med Genet A 2004 124A 318 322 10.1002/ajmg.a.20391 14708108

[B12] Shaikh TH Gai X Perin JC Glessner JT Xie H Murphy K O'Hara R Casalunovo T Conlin LK D'Arcy M High-resolution mapping and analysis of copy number variations in the human genome: a data resource for clinical and research applications Genome Res 2009 19 1682 1690 10.1101/gr.083501.108 19592680 PMC2752118

